# Breaking the rules: is it the neurointensivists’ turn?

**DOI:** 10.1186/s13054-017-1919-3

**Published:** 2018-01-29

**Authors:** Paolo Gritti, Oluwaseun Akeju, Ferdinando Luca Lorini, Andrea Lanterna, Carlo Brembilla, Federico Bilotta

**Affiliations:** 1 Department of Anaesthesia and Critical Care Medicine, Ospedale Papa Giovanni XXIII, Bergamo, Italy; 2 Department of Anaesthesia, Critical Care and Pain Medicine, Massachusetts General Hospital, Harvard Medical School, Boston, MA USA; 3 Department of Neurosurgery, Ospedale Papa Giovanni XXIII, Bergamo, Italy; 4 Department of Anaesthesia and Critical Care Medicine, “Sapienza” University, Rome, Italy

See related research by Cnossen et al. https://ccforum.biomedcentral.com/articles/10.1186/s13054-017-1816-9

We have read with interest the results of the survey by Cnossen et al. [1], in which the authors affirm that substantial variation was found regarding monitoring and treatment policies in TBI patients and intracranial hypertension among 66 European neurotrauma centers. We observe that this result is no different from the conclusion of a similar survey on blood transfusion and coagulation management of TBI patients belonging to the same research group [2]. Moreover, these results agree with another survey on the management of mild TBI patients [3]. The authors’ conclusion shows that, even among high-volume specialized neurotrauma centers, there is a substantial variation in structures and processes of TBI care and a discrepancy between BTF guidelines and reported policies [1–3].

Although this variability between European neurotrauma centers provides an opportunity to study the effectiveness of specific aspects of TBI care and to identify best practices with comparative effectiveness research, the lack of BTF guideline application remains a concern. Compared to a previous study from a decade ago, the data from Cnossen et al. [1] show a reduction in application of the BTF guidelines to 49% and 51% in aggressive and conservative centers respectively. The low adherence to guidelines may explain the variability and heterogeneity of the treatment as we reported recently in the neurointensive care setting of subarachnoid hemorrhage patients due to cerebral aneurysm rupture [4].

While it seems that neurointensivists do not follow the rules, we do not have to forget that this phenomenon has also occurred in other fields of medicine. Arts et al. [5] reported in a recent systematic review that intentional nonadherence to guidelines varied between 8.2 and 65.3%. The same authors concluded that nonadherence is often supported by valid reasons in up to 93.6%. Guideline deviations are intentional, mainly related to contraindications or due to the patient’s decision, and these deviations do not necessarily impact on the quality of care [5].

However, none of the studies analyzed by Arts et al. [5] was carried out in the neuro-ICU setting where the critical situation of the patients, most of them unconscious, postpones any possible consensus decision. Moreover, therapy contraindications disappear when a second-line life treatment is requested. In the hardest of ICU settings, where the patients ‘will disappear’, following guidelines seems to assume a different meaning. We are confident that future studies in the perspective of the CENTER-TBI study can shed light on the actual role of guidelines and protocols in the complex context of neuro-ICUs.

## Abbreviation

TBI: Traumatic brain injury
BTF: Brain Trauma Foundation
CENTER-TBI: Collaborative European Neurotrauma Effectiveness Research in Traumatic Brain Injury
ICU: Intensive care unit
