# Supplementary material for: Breaking the rules: is it the neurointensivists’ turn?
Source: Crit Care. 2018 Jan 29;22:21. doi: 10.1186/s13054-017-1919-3 (PMC5789649; doi:10.1186/s13054-017-1919-3)
Supplement: Related texts

# Reply: Authors' response

**Authors:** Maryse C. Cnossen, Mathieu van der Jagt, Ewout W. Steyerberg, On behalf of the coauthors

We would like to thank Gritti and colleagues for their letter about our recently published manuscript describing considerable variation in monitoring and treatment policies in patients with TBI and intracranial hypertension [1]. This variation probably reflects the lack of evidence [6] and guideline adherence [7] for the treatment of patients with TBI.

Based on Table 1 in the original paper [1], Gritti and colleagues infer that, in comparison to a previous report, guideline adherence to the BTF guidelines has been reduced to 49% and 51% in aggressive and conservative centers, respectively. This statement, however, is incorrect. Of the total sample, the BTF guidelines were used in 49 (74%) centers. Among these 49 centers, 25 (51%) could be classified as ‘aggressive’ and 24 (49%) as ‘conservative’ with regard to ICP management.

Guideline adherence is higher for recommendations based on more robust evidence than for recommendations based on relatively weak evidence [7]. However, subsequent editions of the BTF guidelines have progressively reduced the strength of the recommendations, after reevaluation of the weak evidence. We believe that strengthening the evidence base will result in an improvement in guideline adherence, and subsequently will reduce treatment variation.

Comparative effectiveness research (CER) provides a promising framework for expanding our knowledge of TBI treatment effectiveness. The CENTER-TBI study is currently recruiting approximately 5000 patients from 20 European countries. Once these patient-level data become available, the first step will be to compare the results of the provider profiling questionnaires with the actually observed treatment variation among centers. The next step will be to examine the association between treatment and outcome with CER approaches, to strengthen the evidence underpinning TBI guidelines. A third step will be to develop a tentative set of quality indicators for TBI, which could potentially be used for benchmarking and quality assurance of future TBI care.

As Sackett wrote, “Evidence based medicine is the conscientious, explicit, and judicious use of current best evidence in making decisions about the care of individual patient” [8].

As such, a set of rules to be blindly followed or to be broken has never been proposed. We are confident that detailed large data collection, advanced methodology, and global collaboration with other projects (e.g., TRACK-TBI in the United States) will provide better evidence for grounding better treatments for TBI victims.
